# The Role of Morphine in Animal Models of Human Cancer: Does Morphine Promote or Inhibit the Tumor Growth?

**DOI:** 10.1155/2013/258141

**Published:** 2013-08-28

**Authors:** Sabrina Bimonte, Antonio Barbieri, Giuseppe Palma, Claudio Arra

**Affiliations:** ^1^Istituto Nazionale per lo Studio e la Cura dei Tumori, “Fondazione G. Pascale,” IRCCS, Via Mariano Semmola, 80131 Naples, Italy; ^2^Istituto di Endocrinologia e Oncologia Sperimentale del Consiglio Nazionale delle Ricerche, Dipartimento di Biologia e Patologia Cellulare e Molecolare “L. Califano,” Università degli Studi di Napoli “Federico II,” Via Pansini 5, 80131 Napoli, Italy

## Abstract

Morphine, a highly potent analgesic agent, is widely used to relieve pain and suffering of patients with cancer. Additionally, it has been reported that morphine is important in the regulation of cancerous tissue. Morphine relieves pain by acting directly on the central nervous system, although its activities on peripheral tissues are responsible for many adverse side effects. For these reasons, it is very important also to understand the role of morphine in cancer treatment. The published literature reporting the effect of morphine on tumor growth presents some discrepancies, with reports suggesting that morphine may either promote or inhibit the tumor growth. It has been also demonstrated that morphine modulates angiogenesis which is important for primary tumour growth, invasiveness, and the development of metastasis. This review will focus on the latest findings on the role of morphine in the regulation of cancer cell growth and angiogenesis.

## 1. Introduction

Morphine is used to relieve pains of patients with cancer in terminal phases, in order to improve quality of life [1]. Morphine is an opiate-based drug isolated for the first time in 1803 by Friedrich W. Sertürner [2]. It has been shown that morphine explains its function by acting through opioid receptors, *μ*, *δ*, and *κ*, which are localized in the brain [3, 4]. Morphine relieves pain by acting directly on central nervous system (CNS), although its activity on peripheral tissue leads to many secondary complications, including addiction, respiratory depression, and tolerance. Apart from these severe effects, morphine is still considered the most effective drug clinically available for the management of severe pain associated with cancer [5]. Several experimental studies performed on cancer cell lines and mouse models showed that morphine can also play a role in the regulation of cancer cell growth. Unfortunately, the results obtained by these studies are still contradictory. Some reports demonstrated that morphine inhibited the growth of various human cancer cell lines [6–12] or animal models [13–16]. On the contrary, other studies proved that morphine increased tumor cell growth in *in vivo* [17, 18] or *in vitro* [19] models. According to some studies, morphine at clinically relevant doses stimulated angiogenesis *in vitro* [20] and tumour growth in breast cancer mouse model [21]. It has been demonstrated that morphine modulates angiogenesis which is important for primary tumour growth, invasiveness, and the development of metastasis. For these reasons, there is a dilemma about the effects of morphine on cancer cell growth and angiogenesis. 

This review will focus on the latest findings on the role of morphine in the regulation of cancer cell growth and angiogenesis.

## 2. Morphine Affects Tumor Growth and Apoptosis

The role of morphine in the regulation of tumor cell growth is not yet correctly established. Several xenograft mouse models were generated to study cancer cell growth-promoting or inhibiting effects of morphine. Tegeder et al. [13] generated mouse models of breast cancer by subcutaneous injection of MCF-7 and MDA-MB231 cells in NMRI-nu/nu mice. In these mice, morphine, intraperitoneally injected, significantly reduced tumor growth through a p53-dependent mechanism. Additionally, in these mice, naloxone, an opioid inverse agonist, increased the growth-inhibitory effects of morphine. Similar results were obtained in rat model of colon cancer generated by intraperitoneal injection of colon cancer cells in Fisher 244 rats. In these animals, subcutaneous administration of morphine leads to significant decrease in the hepatic tumor burden. Morphine inhibited not only tumor growth but also metastasis in melanoma mouse model generated by subcutaneous injection of B16-BL6 cells into the hind paws of C57BL mice [15]. Another group, demonstrated that morphine inhibited tumor metastasis formation when it was administered intraperitoneally in mouse model of colon cancer [16]. On the contrary, several experimental studies demonstrated that morphine increased tumor growth. Gupta et al., in orthotropic mouse model of breast cancer obtained by injection of MCF-7 cells into the mammary fat pads of nude mice, demonstrated that morphine, in clinically relevant doses, increased tumor growth. This was associated with increased angiogenesis and inhibition of apoptosis and promotion of cell cycle progression [20]. In this study, it was also reported that naloxone itself had no significant effect on angiogenesis. Our preliminary data, obtained by *in vitro* and *in vivo* experiments using MDA.MB231 breast cancer cells, seems to validate this hypothesis (Bimonte et al., unpublished data). According to these results, in another study, it was demonstrated that morphine, subcutaneously administrated in mice, increased the tumor growth in mouse model of leukaemia and sarcoma. In these mice, morphine played also a general immunosuppressive role [22]. 

These contrasting results are probably associated with different concentration and/or time of administration of morphine. In fact, *in vitro* and *in vivo* studies demonstrated that tumor-enhancing effects with morphine occur after administration of low daily doses or single dose of morphine [23], while tumor suppression occurs after chronic high doses of morphine [11, 15, 16].

It has also been demonstrated that the *μ*-opioid receptor, by which morphine exerts its action, directly regulates tumor growth and metastasis. On the basis of these results, different mechanisms of opioid receptor-mediated influence of morphine on tumor growth have been proposed. Morphine, as mentioned above, after binding to the *μ*-opioid receptor, regulates cell cycle progression by stimulating mitogen-activated protein kinase (MAPK)/extracellular growth factor (Erk) pathways [20]. Alternatively, morphine can mediate apoptosis by activating phosphatidylinositol 3-kinase (PI3K)/protein kinase B (Akt) pathway [24]. Additionally, morphine, by the upregulation of urokinase plasminogen activator (uPA) expression, induces metastasis formation [25], while, by transactivation of VEGF receptor, it induces angiogenesis [26]. Finally, morphine affects also the function of T lymphocytes, leading to immunosuppression [27]. 

It has been proposed that morphine plays also a role in tumor apoptosis. Apoptosis is a form of cell death in which a programmed sequence of events leads to the elimination of cells without releasing harmful substances into the surrounding area. It is noted that apoptosis is regulated by two pathways: the mitochondrial-mediated pathway (intrinsic) [28] and death receptor-mediated pathway (extrinsic) [29]. It is noted that in cancer cells apoptosis is deregulated, and this leads to quick proliferation and tumor growth [30, 31]. Morphine was shown to induce apoptosis of macrophages, T lymphocytes, and human endothelial cells [32, 33]. Experiments performed on human tumor cell lines demonstrated that morphine in high concentration induces apoptosis and inhibits cancer cell growth by activation of different signal pathways involving caspase 3/9, cytochrome c, and sigma-2 receptor. On the contrary, it has been demonstrated that morphine can inhibit apoptosis. Additionally in SH-SY5Y cells, morphine has antiapoptotic effect by antagonizing doxorubicin [34]. These discrepancies, also in these cases, are associated with different cell line tumor type used and/or *in vivo* dose/time of morphine administrated. 

## 3. Morphine Regulates Angiogenesis and Metastasis Formation

Recent data demonstrated a role of morphine in angiogenesis. Angiogenesis is required for invasive tumor growth and metastasis and represents an important point in the control of cancer progression. Proangiogenic activity of morphine was demonstrated in the MCF-7 breast cancer model. In these mice, morphine at clinically relevant concentrations enhanced tumor neovascularization [20]. In an animal model of hormone-dependent breast cancer, it has also been demonstrated that morphine promoted activation of vascular endothelial growth factor (VEGF) receptor and increased metastasis [21, 27]. It has been proposed that morphine explains its proangiogenic activity by the stimulation of mitogen-activated protein kinase (MAPK) signalling pathway via G protein-coupled receptors and nitric oxide (NO). Alternatively, several *in vivo* studies provided evidence that morphine can induce tumor growth by the upregulation of cyclooxygenase-2 (COX-2) [35–38] and/or prostaglandin E2-mediated stimulation of angiogenesis [39–42]. On the contrary, several *in vivo *and *in vitro* studies demonstrated that morphine can inhibit angiogenesis by the regulation of different pathways [8, 32, 43, 43–50]. These different results can be due to different experimental conditions (cell line tumor type used and/or dose/time of morphine). Morphine plays a role not only in tumor cell growth but also in metastasis formation, which is the main process related to most cancer deaths and failure in cancer treatment [51, 52]. The process which leads to metastasis formation initiated with migration of cancer cells through the extracellular matrix (ECM). Both pro- and antimigratory effects have been reported for morphine. Specifically, it has been shown that morphine significantly reduces the adhesion, invasion, and metastasis of metastatic colon 26-L5 carcinoma cells [16], by the regulation of matrix metalloproteinases (MMPs). On the contrary, morphine can promote invasion, metastasis formation and migration of cancer cells by the upregulation of MMPs in breast and lung cancer [52, 53]. Finally, in MCF-7 breast cancer cells [50, 54] and in H729 cancer cells [55], morphine treatments lead to the upregulation of urokinase plasminogen activator (uPA) which promotes migration of cancer cells through the ECM.

## 4. Conclusions

Several studies provided evidence that morphine can affect tumor growth by acting with different mechanisms, including tumor cells or endothelial cells or growth factors secreted by meditation of CNS. Unfortunately, the results obtained from both *in vitro* and *in vivo* studies are so far conflicting. Some reports suggested that morphine may promote the tumor growth by inhibiting apoptosis and by promoting angiogenesis and migration of tumor cells. On the contrary, it has been demonstrated that morphine may also exert proapoptotic and antiangiogenic effects. These different results can be associated with the different doses of morphine administrated, with different models used and different cancer. For these reasons, it is very important for the management of severe pain associated with cancer to consider accurately the dose and route of administration of morphine. Further studies will be necessary to establish if morphine is an inhibitor of tumor growth or whether it promotes cancer. 
